# The Comparison of the Plasma Levels of the Lead Element in Patients with Gastrointestinal Cancers and Healthy Individuals

**DOI:** 10.31557/APJCP.2019.20.9.2639

**Published:** 2019

**Authors:** Masoudreza Sohrabi, Zahedin Kheiri, Ali Gholami, Mehran Haghighi, Fahimeh Safarnejad Temeshkel, Mahmood Khoonsari, Majid Reza Adelani, Amir Hossein Mirhosseini, Melika Sohrabi, Azam Rezaei Farimani, Farhad Zamani, Hossein Ajdarkosh, Amir Hossein Faraji

**Affiliations:** 1 *Gastrointestinal and Liver Diseases Research Center (GILDRC), Firoozgar Hospital, Iran University of Medical Sciences,*; 4 *Department of Analytic Chemistry, Faculty of Basic Sciences, Tehran Islamic Azad University, North Branch, Tehran,*; 2 *Department of Internal Medicine, Arak University of Medical Sciences, Arak,*; 3 *Noncommunicable Diseases Research Center,*; 5 *Department of Basic Medical Science, Neyshabur University of Medical Sciences, Neyshabur, Iran. *

**Keywords:** Lead, cancer, Gastrointestinal, trace elements

## Abstract

**Back ground and Aim:**

Heavy metals are considered as risk factors in the development of some types of cancers. In this context, the lead (Pb) along with its biological impacts on the human body has raised significant concerns in public health. The aim of this study was to compare the plasma levels of the lead element in patients with gastrointestinal (GI) cancer and healthy subjects to examine whether this element has a role in the susceptibility of cancer.

**Methods:**

In a case-control study conducted between March 2016 to February 2017, the plasma levels of the lead were assessed. One-hundred patients with upper and lower GI cancers, as well as one-hundered healthy subjects who were age- and sex-matched participated in our study. A classic flame atomic absorption spectroscopy (FAAS) method was employed for the determination of the lead element in plasma levels of all subjects.

**Results:**

The mean age of patients was 53.8±10.6 years old. The patient group consisted of 51 male and 49 female patients. The results showed that the concentrations of Pb were lower than the defined toxic levels. The comparison of the mean levels of Pb between the case and control groups revealed that there was no statistically significant difference even when the gender, age, and history of smoking were included in the statistical analysis. Our findings showed that the concentration of Pb is significantly associated with the type of cancer (p<0.003) and the location of the tumor (whether upper or lower tract was affected) (p<0.003).

**Conclusion:**

Lead may contributes to the pathology and progression of GI cancers but we can not conclude that it involved in the causation or susceptibility of healthy individuals to develop GI cancer.

## Introduction

In recent years, the incidence of cancer, especially gastrointestinal cancer (GI) is sharply increased in developing countries that it is thought the interplay between the environmental factors and genetic susceptibility plays a crucial role in the development of GI cancer (Koochak et al., 2016; Torre et al, 2015;Hemmasi et al., 2015; Anton-Culver et al., 2016; Rakhshani et al., 2014). Among environmental factors, heavy metals are involved in the causation and progression of many types of malignancies (Navarro Silvera and Rohan, 2007; Galanis et al., 2009). The lead element (Pb) is one of the heavy metals which is broadly applied in numerous industrial products. The routes of exposure to Pb can occur via ingestion, inhalation, and dermal contact. Upon the entrance of Pb into the human body, it mediates some toxic reactions within the body and affects some organs such the central nervous system, reproductive, hematopoietic, and cardiovascular systems (Iarc et al., 2006; Fang et al., 2014). The source of environmental contamination varies as automobile exhaust, industrial wastewater, solid waste, cosmetic and paint products could contribute to the release of Pb into the environment. During the 1990s, the Pb toxicity in pediatric populations raised some concerns in the United States (US) and China (Li et al., 2014). They indicated that Pb toxicity could lead to intellectual and cognitive impairments and cause behavioral aberrations in children. It has been reported that another source of Pb contamination may stem from opium usage. In recent years, in some part of the Middle East where the consuming of opium is very common, the reports of Pb toxicity has tremendously increased. The lead element elevates the weight of opium without any change in its color or smell of the substance (Kamangar et al., 2014; Naghibzadeh Tahami et al., 2014). Hence, inhalation or ingestion of these products may cause Pb toxicity, and its side effects might influence the functionality of some organs in the human body. To date, there no definite cut off for Pb poisoning; thus, the intoxication may occur even at low concentrations of Pb (AAPC, 2005). According to reports of the American Academy of Pediatrics, between 2007 and 2010, approximately 2.6% of pre-school children in the US had plasma levels of Pb higher than 0.05 µg/ml which caused neurobehavioral problems in children. However, there was no linear correlation between the concentrations of Pb and the severity of side effects in children exposed to Pb (AAPC, 2016). There is no cure for individuals intoxicated with Pb (Garcia-Leston et al., 2010; Silbergeld et al. , 2000). Although human studies have not definitely confirmed the link between cancer and Pb toxicity, especially GI cancer, but a number of animal studies have suggested that exposure to inorganic Pb increases the risk of kidney, lung, brain, and hematopoietic cancers(Saghiri et al., 2016; Chan et al., 2015). Despite the increasing knowledge about the genotoxicity of Pb, the mechanism underlying this phenomenon is still opaque. It has been proposed that Pb may induce inflammation, oxidative stress, and inhibit DNA repair, as well as causing genetic mutations in human beings when exposed to Pb for a long-term period. Previous studies demonstrated that Pb is involved in some signaling pathways which contribute to inflammation and neutrophil activity and consequently can cause cellular damages (Kim et al., 2015; Lin et al., 2015; Garcia-Leston et al., 2010). Therefore, the abnormal concentrations of Pb may have a marked impact on cell biology and could be considered a carcinogen compound. In this case-control study, we evaluated the concentrations of Pb in plasma samples of patients with gastrointestinal cancers in comparison with healthy subjects.

## Materials and Methods

*Study design and sample collection*

This case-control study was conducted in Firoozgar Hospital affiliated to the Iran University of Medical Sciences. Data were collected from March 2016 to February 2017. One-hundred patients diagnosed with gastrointestinal cancers, by histopathological examinations, were enrolled in this study. The same number of healthy individuals who were age- and sex-matched were also included in our study as a control group. The exclusion criteria for the selection of patients with GI cancers were as follows; disagreement of patients, history of malignancies other than gastrointestinal cancers, history of metabolic or nutritional disorders, the regular use of a particular medication/hormones/chelators, receiving chemotherapy or radiations. The process of the selection of healthy individuals was based on those subjects referring to the hospital for the annual check-up, and they were devoid of any GI problems. The same exclusion criteria were met for the selection of the control group except for “receiving chemotherapy and radiation” that was not applicable and meaningful for healthy individuals.

*Demographic and clinical characteristics of participants*

A questionnaire including demographic (age, sex, and history of smoking) and clinical (the type and location of GI cancers) was given to each patient or healthy individual.

*Diagnosis and classification of GI cancers*

GI cancers were divided into four categories including gastric, esophageal, colon, and rectosigmoid cancers. Also, the site of GI tumors was assigned into two classifications according to their anatomical location namely, lower and upper gastrointestinal cancers. Lower gastrointestinal malignancies included colon and rectosigmoid cancers and upper gastrointestinal cancers consisted of the esophagus and gastric cancers. 

*Measurement of plasma Pb*

*Sample Preparation*

Acid digestion was employed for the preparation of the blood samples. Venous blood samples were obtained from all participants and immediately poured in heparin-containing tubes. Samples were agitated by the hand and centrifuged at 2,000 rpm for 10 min. The plasma was separated and stored at -70°c until analyzed. At the laboratory, 3 ml of samples were digested with 65% HNO_3_- 30% H_2_O_2_ mixture (10:1 v/v) and heated until dense white fumes appeared. Samples were cooled down to room temperature, filtered, and then diluted to a proper volume with double distilled water. The final solution was injected to the FAAS in order to determine the concentration of Pb in plasma specimens.

**Table 1:** Descriptive Statistics of Blood Pb Concentration (µg/ml) in Patients and Healthy Controls Based on Different Characteristics of Study Population

Variables			N	Median	Mean	SD	Minimum	Maximum
Gender	Male	Case	51	0.022	0.14	0.27	0.04	1.11
		Control	51	0.039	0.04	0.04	0	0.161
	Female	Case	49	0.028	0.15	0.35	0	2.08
		Control	49	0.039	0.04	0.04	0	0.161
Age categories	<50 y	Case	36	0.03	0.2	0.43	0	2.08
		Control	36	0.039	0.04	0.04	0	0.161
	50-60 y	Case	34	0.02	0.13	0.21	0	0.92
		Control	34	0.044	0.04	0.03	0	0.106
	>60 y	Case	30	0.02	0.08	0.21	0	1.11
		Control	30	0.011	0.03	0.04	0	0.161
History of Smoking	No	Case	36	0.022	0.14	0.39	0	2.08
		Control	45	0.039	0.04	0.03	0	0.106
	Yes	Case	64	0.028	0.14	0.26	0	1.11
		Control	55	0.033	0.04	0.04	0	0.161
Type of Cancer	Esophagus	Case	13	0.033	0.19	0.32	0	1.11
	Gastric	Case	37	0	0.13	0.39	0	2.08
	Colon	Case	34	0.028	0.14	0.27	0	0.92
	Rectal	Case	16	0.061	0.14	0.15	0.017	0.46
Location of cancer	Upper	Case	50	0.011	0.14	0.37	0	2.08
	Lower	Case	50	0.033	0.14	0.24	0	0.92
Total		Case	100	0.14	0.03	0.31	0	2.08
Total		Control	100	0.039	0.04	0.04	0	0.161

**Table 2:** Comparison of Pb Concentration (µg/ml) in Blood of Cases and Healthy Controls with Use of Wilcoxon Rank-Sum and Kruskal-Wallis Rank Tests According to Different Characteristics of Study Population

	Variable		Median	Z or X^2^	*P-value*
Case group	Gender	Male	0.022	0.695	0.49
		Female	0.028		
Control group		Male	0.039	0.362	0.71
		Female	0.039		
Case group	Age categories	<50	0.03	1.95	0.38
		50-60	0.02		
		>60	0.02		
Control group	Age categories	<50	0.039	5.92	0.051
		50-60	0.044		
		>60	0.011		
Case group	History of Smoking	No	0.022	-0.592	0.55
		Yes	0.028		
Control group	History of Smoking	No	0.039	0.587	0.56
		Yes	0.033		
Case group	Type of Cancer	Gastric	0.000	13.68	0.003
		Esophagus	0.033		
		Colon	0.028		
		Rectal	0.061		
Case group	Location of cancer	Upper	0.011	2.93	0.003
		Lower	0.033		

**Table 3:** Comparison of Pb Concentration (µg/ml) in Blood of Cases and Healthy Controls with Use of Wilcoxon Matched-Pairs Signed- Ranks Test According to Different Characteristics of Study Population

variable		Median		Wilcoxon Z test	P value
		Case	Control		
Gender	Male	0.022	0.039	-0.966	0.33
	Female	0.028	0.039	-0.443	0.66
Age categories	<50	0.03	0.039	-1.078	0.28
	50-60	0.02	0.041	-0.385	0.7
	>60	0.02	0.011	-0.382	0.7

*Instrument*

The flame atomic absorption spectroscopy (FAAS) method, using the Varian model spectra AA-240 (Mulgrara, Victoria, Australia) was used to assess the levels of Pb in plasma samples. An Alpha silver heater and Mettler College 150 balances were used. The parameters for the instrument were set based on the manufacturer’s recommendations.

*Reagents*

All reagents and solvents were procured from Merck Company (Germany). Nitric acid (65%) and double distilled water were used to wash the glassware. Nitric acid (65%) and hydrogen peroxide (30%) were also employed for the digestion procedures. In the next step, standard solutions for the measurement of Pb were prepared in a range of 0.05-3 µg/ml. The calibration curve was obtained using the analysis of the standard solutions at a wavelength of 217 nm, HC Lamp Current (5mA) and Slit width (1 nm).

*Statistical analysis*

The analysis of the data obtained in this study was performed by the SPSS software version 22 (SPSS, Chicago, IL). The Shapiro-Wilk test was used to determine whether the data were normally distributed. The Wilcoxon and Kruskal-Wallis tests were used for the comparison of the levels of Pb between the case and control groups where appropriate. The confidence interval was set at 95%, and the difference between the two groups was considered statistically significant if the p-value was less than 0.05. The age of participants was categorized into three groups as follows; <50 years, 50-59 years, and ≥60 years. Smoking history was expressed as the Yes/No answer. 

*Ethical considerations*

This study was approved by the Ethics Committee of the Iran University of Medical Sciences, Firoozgar Hospital. Before the commencement of the study, all subjects were given informed consents. The experimental protocol was designed and carried out according to the principles of the Helsinki Declaration.

## Results

Among one-hundred patients with GI cancers, 51 patients were male possessing the mean age of 53.8±10.6 years old. More than two-thirds of the patients suffered from gastric (37 patients) and colon cancers (34 patients). The same number of healthy individuals was employed in the control group which was age- and sex matched with the case group (Table 1). 

In this study, half of GI cancers were related to the lower gastrointestinal tracts. The plasma concentrations of Pb in patients with cancer were presented in Table 1. As shown in Table 1, although the concentration of plasma Pb in the case group was not significantly correlated with gender (p=0.49), age (p=0.38), and history of smoking (p=0.55), the levels of plasma Pb was statistically associated with the types (p=0.003) and the tumor site of GI cancers (p=0.003). 

Tables 1 and 2 also showed that similar findings were obtained from the comparison of plasma levels of Pb with gender (p=0.71), age (p=?), and history of smoking (p=0.56) in healthy subjects. Finally, as indicated in Table 3, no significant difference was observed in the concentration of Pb between the case and control group when gender, age, and history of smoking were considered (all p-values >0.05).

## Discussion

In the present study, we attempted to determine the association between the plasma concentrations of Pb and the susceptibility of GI cancers. There was no statistically significant difference in plasma levels of Pb between the healthy subjects and patients with GI cancers. However, there was a significant correlation between the concentration of Pb and the type and tumor site of GI cancers.

The National Institute for Occupational Safety and Health (NIOSH) indicated that the normal plasma levels of Pb were defined as 0.05 µg/ml for adults (Alarcon et al., 2015). Besides, Pb intoxication-induced the central nervous system dysfunction has been reported when the plasma levels of Pb is in a range of 0.82-1.44 μg/ml. Additionally, the toxic levels of Pb for the gastrointestinal tract dysfunction has been defined in a range of 0.41-0.82 μg/ml (Chan et al., 2015). In this study, the concentration of Pb did not reach the above ranges mentioned earlier. But, it seems that cancer-prone tissues such as the GI tract have high sensitivity to Pb-induced damage even at lower concentrations. In this context, animal studies demonstrated that exposure to inorganic Pb causes kidney, lung, and brain cancers (Rousseau et al., 2007; Chan et al., 2015; Wynant et al., 2013). Furthermore, the tissue concentration of Pb could be regarded as an issue that needs to be more taken into account. Although it is not clear whether the low levels of Pb could contribute to the development of GI cancers, it is likely that Pb might trigger, along with other carcinogen agents, environmental or genetic factors which are thought to be involved in the etiology of GI cancers (Sohrabi et al., 2017).

International Agency for Research on Cancer (IARC) has shown that Pb might be carcinogenic for the human body (Kim et al., 2015). There are few studies on GI cancers and plasma levels of Pb. In this line, some studies performed on labors who were exposed to Pb suggested that Pb may be involved in the development of specific types of malignancies such as lung and gastric cancers (Wingren and Axelson, 1993; Gerhardssonet et al., 1995; Gerhardsson et al., 1986; Pukkala et al., 2009). In the year of 2008, Khorasani et al., in a case-control study, measured the plasma levels of Pb in patients with gastric cancer and healthy subjects. They found that the plasma levels of Pb were higher in patients with gastric cancer compared with the control group.

Furthermore, Rousseau and colleagues showed that men workers who were exposed to had a higher risk of developing gastric cancer compared with the general population; however, the risk of other types of cancer was not significantly higher in male labors when compared with the other people. In contrast, Lam et al. indicated that there was no significant association between the exposure of male workers to Pb and the risk of gastric cancer. In the present study, we indicated that the concentrations of Pb in patients with lower GI cancers were higher than patients with upper GI cancers. Also, our previous study showed that concentrations of Pb in patients with colon cancer were higher in cancerous tissue as compared with non-cancerous tissues (Sohrabi et al., 2017). Generally, the exposure to Pb occurs in the upper GI tract since the main route of Pb contamination is mediated by ingestion and inhalation. Hence, it would be plausible that the rate of upper GI cancers is higher than the lower GI cancers. It is alleged that the blood circulation can transfer the lead element to other organs within the human body and therefore, the systemic circulation plays a central role in the susceptibility of lower GI cancers in individuals exposed to Pb. It seems that Pb is idiopathically accumulated in the lower GI tract and increases the risk of GI cancers in individuals prone to develop GI cancer. To date, though there is no consensus on the carcinogenicity of Pb in the literature, the detrimental effects of the lead element is undeniable on the biological reactions occurred within the human body such as the inhibition of DNA repair.

In 2006, Dobrakowski and collogues exhibited that chronic and sub-chronic exposure to Pb may increase inflammation activity and induce pro-inflammatory cytokines and non-enzymatic antioxidants, such as bilirubin. Regardless of the free radical scavenging role of bilirubin, higher levels of bilirubin are capable of inhibiting the biosynthesis of the heme molecule (Dobrakowski et al., 2014; Annabi Berrahal et al., 2007; Aziz et al., 2018). Pb can also influence the cellular inflammatory response and enhance the angiogenesis process. Moreover, this element has indirect genotoxic potentials when administered in combination with other DNA-damaging agents, suggesting a possible co-carcinogenic effect (Rousseau et al., 2007; Lin et al., 2015). Therefore, it would be expected that Pb can trigger the cellular damages in the lower tract of GI cancers after the absorption via the different cell signaling pathways.

In conclusion, the concentration of Pb did not exceed the toxic levels in plasma levels of either patients or healthy individuals. However, we could not rule out the side effects of Pb on DNA and inflammation. In the present study, it was shown that the concentration of Pb was linked with the type and tumor site (upper and lower GI cancers). It appears that the rout of exposure to Pb does not play a significant role in the development of GI cancers.
